# Organoid Models of Human Endometrial Development and Disease

**DOI:** 10.3389/fcell.2020.00084

**Published:** 2020-02-18

**Authors:** Youssef Hibaoui, Anis Feki

**Affiliations:** ^1^Department of Genetic Medicine and Development, University of Geneva Medical School, Geneva, Switzerland; ^2^Service de Gynécologie Obstétrique, HFR Fribourg - Hôpital Cantonal, Fribourg, Switzerland

**Keywords:** endometrial organoids, iPSCs, endometrial development, embryonic implantation disease modeling, infertility, pregnancy disorders, endometriosis, cancer

## Introduction

The endometrium is a complex multicellular tissue lining the uterus that plays a crucial role in human reproduction. This multi-layered organ is composed of a functionalis layer, adjacent to the uterine lumen and a basalis layer, attached to the myometrium. These layers comprise several cell types including luminal and glandular epithelial cells, endometrial stromal cells, immune cells and vascular cells forming the spiral arterioles (Kobayashi and Behringer, 2003; Roy and Matzuk, 2011; Hart, 2016). This unique tissue undergoes remarkable dynamic remodeling orchestrated by menstrual repair, proliferation and differentiation processes, which are carefully controlled by female sex hormones during the menstrual cycle. The remodeling requires a delicate interplay of cellular and molecular events that enable the endometrium to be receptive for embryo implantation. During the proliferative (or follicular) phase, both the endometrial glands and stroma of the functionalis layer proliferate in response to rising estrogen levels coming from the ovarian follicular. Consequently, the thickness of the endometrium increases. Then, the functionalis layers maturate during the secretory (or luteal) phase with the concomitant presence of secretory and ciliated cells in response to progesterone. In the absence of conception, this tissue sheds before regenerating for a subsequent menstrual cycle (Kobayashi and Behringer, 2003; Roy and Matzuk, 2011). Defects in endometrium remodeling and function can lead to the development of various types of disorders that affect considerable numbers of women. These includes infertility, pregnancy disorders, endometriosis and endometrial cancers (Hart, 2016). For instance, endometriosis a condition in which endometrial tissues proliferate outside the uterus leading to pelvic pain, excessive bleeding and infertility affects between 10 and 15% of all women of reproductive age (Giudice and Kao, 2004; Vercellini et al., 2013). Endometrial cancer, the most common malignancy of the female genital tract, affects ~3% of women, with the highest rates registered in North America and Europe (Lortet-Tieulent et al., 2017; Urick and Bell, 2019).

Even if progress has been made in the knowledge of the endometrium structure and function, little is known regarding the molecular and the cellular mechanisms involved in this dynamic remodeling in both physiological and pathological conditions. The main obstacle of such studies is the lack of accurate models. Many insights have been provided thanks to studies using mouse models. However, these models do not accurately recapitulate the specificities of human endometrial development and function. As a matter of fact, endometrium decidualization in mice and humans are quite different. Decidualization of the endometrium occurs in rodents exclusively when there is an incoming embryo or in response to mechanical injury, whereas in humans the endometrium undergoes decidualization in a cyclic manner, regardless of the presence of an embryo (Gellersen et al., 2007; Peterse et al., 2018). Therefore, findings obtained with these animals often cannot be directly translated to humans. Immortalized or carcinoma-derived cell lines, such as Ishikawa cells (derived from epithelial endometrial cells) or St-T1b cell (derived from stromal endometrial cells) are also available for *in vitro* investigations. While these cells are easily cultured for long periods (Table 1), there are general limitations of using such cell lines including their genetic background, the potential changes acquired following transformation and during their establishment in culture (Mannelli et al., 2015). Alternatively, primary endometrial cells can be isolated, but these are difficult to maintain and expand in long term culture. Most of these cells lose their phenotype and hormone responsiveness in culture (Mannelli et al., 2015). Moreover, two dimensional (2D) cell cultures do not faithfully mimic *in vivo* three dimensional (3D) structure and function. These limitations prompted researchers to find novel strategies to model human endometrium. To that end, 3D-culture methods, namely organoids hold promise as models to better mimic *in vivo* human endometrium.

**Table 1:** Comparative advantages and drawbacks of the current *in vitro* endometrial models.

	**Immortalized human endometrial cell lines**	**Organoid culture of human endometrium**	**Endometrial cells derived from human iPSCs**
Rate of initial establishment	Moderate	Moderate	Moderate
Derivation time	2–4 months	~1 month	2–4 months (including differentiation)
Maintenance in culture	Easy	Not easy	Not easy
Required expertise	Low	High	High
Expansion	Very high	High (but limited)	Very high
Cost	Low	High	High
Endometrial development and disease modeling	No	Yes	Yes
Genetic manipulation	High	High	High
Possibility to generate isogenic controls	Yes	Yes	Yes
Microenvironment of the *in vivo* tissue	No	Yes	Possible using co-culture

## Organoids Derived From Human Primary Endometrium

**Figure 1:**
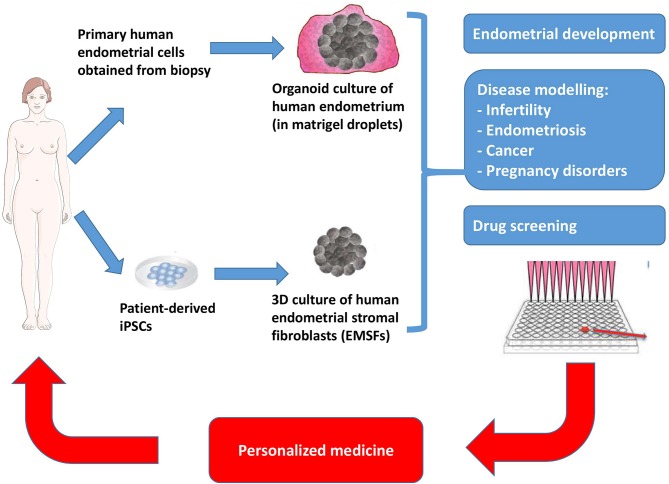
Schematic representation of 3D-culture technology for generating endometrial organoids from patient biopsy, and for differentiating patient-derived iPSCs into endometrial stromal fibroblasts (EMSFs). These 3D-culture methods will lead to valuable clinical insights thanks to their potential applications in endometrial development, disease modeling and drug screening. Ultimately, these methods hold special promise for the development of personalized medicine approaches.

Organoids can be defined as 3D *in vitro* tissue models that recapitulate many of the physiologically relevant properties and features of the *in vivo* tissue. Numerous studies have succeeded to derive organoids from various primary tissues such as intestine, liver, pancreas (reviewed in Clevers, 2016). The recent discovery that human endometrial organoids can be generated from primary endometrial cells has opened up new possibilities to investigate biological processes involved in human pregnancy, disease modeling and testing therapeutic compounds for clinical applications (Boretto et al., 2017; Turco et al., 2017). To generate human endometrial organoids, primary endometrial tissue samples are dissociated with enzymatic procedures (collagenase and/or dispase) and resuspended into Matrigel droplets in a defined medium that was shown to promote organoid formation and maintenance from primary tissue or iPSC-derived cells (Figure 1). The define medium includes activators of WNT signaling (WNT ligands and R-respondin-1), growth factors (EFG, FGF10), TGFβ inhibitors (A83-01), BMP inhibitor (Noggin) and nicotinamide. Importantly, these organoids can be generated from endometrium biopsies obtained throughout the menstrual cycle phases, as well as from endometrium from pregnant and post-menopausal women. Moreover, these organoids can be extensively passaged (every 7–10 days for more than 6 months in culture) while maintaining genetic and phenotypic stability (Turco et al., 2017). This provides opportunity for cryopreserved bio–banking of these endometrial organoids and easily accessible resource for future investigations.

Endometrial organoids have been shown to recapitulate many features of human endometrium. First, genomic analysis through RNA sequencing reveals that these endometrial organoids cluster more closely to glands than to stroma, which recapitulates the molecular signature of the endometrial glands *in vivo*. Second, these organoids respond to sex hormones in a similar manner to that expected *in vivo*. Organoids treated with progesterone exhibited characteristics of the endometrium in its secretory phase, with an increased folding of the glands with the presence of subnuclear vacuolation in the columnar cells. In line with this, endometrial organoids have the ability to generate the two main endometrial cell types: (i) the secretory cells (progestogen-associated endometrial protein (PAEP) positive) that are present in the *in vivo* secretory endometrium, and (ii) and ciliated cells (acetylated-α-tubulin positive) that are present *in vivo* in the uterine luminal epithelium and in superficial glands (Boretto et al., 2017; Turco et al., 2017). By contrast, organoids treated with estrogen displayed morphologic characteristics of the endometrium during the proliferative phase, as revealed by the presence of pseudostratified glandular epithelium. Cells positive for the TRH (Thyrotropin-releasing hormone) marker were also found in organoids treated with estrogen (Boretto et al., 2017; Turco et al., 2017).

## Endometrium 3D Culture Derived From Human Induced Pluripotent Stem Cells

Soon after the generation of organoids from human primary endometrium, a method for culturing human endometrial stromal fibroblasts (EMSFs) from induced pluripotent stem cells (iPSCs) was published by Miyazaki et al. (2018) (Figure 1). Using embryoid body culture conditions, iPSCs were successively directed into intermediate mesoderm (IM, after 4 days of differentiation) and the Müllerian duct (MD, after 8 days of differentiation), a tissue that gives rise to the female reproductive tract including the uterus, the oviduct and the upper vaginal canal. Thereafter, MD cells were further differentiated into endometrial stromal fibroblasts (EMSFs) using 5′-aza-2′-deoxycytidine (5aza2), CHIR99021, 17β-estradiol (E2), FGF9, and PDGF-BB for another 6 days. As expected for EMSFs, these cells expressed the critical endometrial markers including HOXA10, HOXA11, and PGF genes/proteins (Miyazaki et al., 2018). Interestingly, iPSC-derived EMSFs undergo decidualization in response to an 8 days-treatment with a hormonal-based cocktail containing E2, progestin and 8-bromoadenosine 3′,5′-cyclic adenosine monophosphate (cAMP), recapitulating hormone responsiveness of the endometrial stroma. Markers for decidualization such as *FOXO1, HAND2, IGFBP1*, and *PRL* were all found to be up-regulated using this approach. Moreover, RNA sequencing analysis of these iPSC-derived EMSFs confirmed a transcriptional signature which recapitulated endometrial stroma (Miyazaki et al., 2018).

## Advantages and Applications of Endometrial Organoids

It is now widely accepted that 3D organoids more accurately mimic the structural and the functional properties of the *in vivo* tissue compared with its 2D cell culture counterparts. One reason for this is the ability of organoids to recapitulate the physiological, biomechanical, and biochemical microenvironments of the *in vivo* tissue, which is simply not possible in the case of 2D cell culture growing in a homogeneous monolayer. As such, organoids allow cell-cell interactions and cell-extracellular (ECM) matrix interactions in all the three dimensions, interactions which are very limited, if not impossible, in 2D cell culture (Table 1).

A key advantage of endometrial organoid model is the ability to genetically modify the cells, which represents a significant benefit compared with the time and the energy required to create for instance a knock-out mouse model (Table 1). These organoids can be manipulated genetically through various means, including both viral (e.g., lentiviral and adeno-associated viral vectors) and non-viral (e.g., electroporation) approaches, lending itself favorably to gene editing and transposon-based gene modification strategies. In this regards, CRISPR/Cas9, the most attractive gene editing technique, has enabled researchers to efficiently manipulate genomic sequences in organoids derived from primary tissue or from iPSCs, repairing for instance putative genetic mutations in patient-derived iPSCs or by introducing genetic mutations in healthy wild type cells (Matano et al., 2015).

Perhaps the most obvious application of endometrial organoids is as an *in vitro* model system for researchers studying endometrial development. As these cultures can incorporate most of the cell types present in the *in vivo* tissue and recapitulate many of the structural and functional properties of the *in vivo* tissue, endometrial organoids constitute a valuable tool for the investigation of the changes and events that occur during the menstrual cycle and during early pregnancy following the establishment of the placenta. Until now, it was not possible to adequately capture these events in a woman. Moreover, endometrial organoids may be an alternative to the existing models used for the investigation of embryo-endometrium interactions (reviewed in Weimar et al., 2013). It allows to study of the initial stages of human embryo implantation *in vitro* and should provide a better understanding of the mechanisms underlying human embryo-endometrium cross-talk. Such studies are a prerequisite for the improvement of assisted reproduction outcomes and for the prevention of early pregnancy loss.

In the last decade, organoid culture has been widely used to model human disease *in vitro* (the so-called “disease in a dish”). Recent progress in generating endometrial organoids from either primary biopsy or iPSCs hold great promise to model endometrial disorders such as infertility, pregnancy disorders, endometrial cancers, endometriosis, Asherman syndrome, and possibly others (Figure 1). In this respect, Turco and colleagues have succeeded to derive organoids from endometrial cancer from post-menopausal women (Turco et al., 2017). These organoids phenocopied the morphology of the primary tumor from which it was derived (FIGO grade I endometrioid carcinoma with predominant gland growth, nuclear pleomorphism, and disorganized epithelium with irregular basement membrane), supporting the idea that this model recapitulates the histological organization and phenotype of the endometrial carcinoma. Also, this model allows the comparison of the endometrial carcinoma tissue with the normal adjacent endometrium providing an isogenic control tissue, without the biological “noise” that could result from the variability of an individual's genetic background (Turco et al., 2017). More recently, Boretto et al. derived organoids from patients with low- to high grade endometrial cancers. Interestingly, these organoids capture accurately cancer subtypes, recapitulate disease phenotype and display patient-specific drug responses (Boretto et al., 2019). Patient-derived endometrial organoids can also be exploited for modeling endometriosis, a disease that affects between 10 and 15% of all women of reproductive age and 70% of women with chronic pelvic pain (Giudice and Kao, 2004; Vercellini et al., 2013). When one considers that there is no curative solution for endometriosis (Fadhlaoui et al., 2014), it is hoped that endometrial organoids may help to accelerate the development of novel therapeutics in this area of research.

As alluded to above, a promising avenue for use of endometrial organoids in translational research is the possibility to identify novel therapeutic targets and at the same time, to perform screens of molecules for endometrial diseases (Figure 1). Endometrial organoids derived from primary biopsies or from iPSCs can provide a more relevant model for high throughput drug screening than immortalized or carcinoma-derived cell lines. Also, patient-derived endometrial organoids offer a powerful model for predicting efficacy and safety at preclinical stages. In fact, toxicology testing is one of the most attractive uses of diseased endometrial organoids, given that it is possible to generate healthy controls (matched isogenic and non-isogenic), as well as cells and tissues that are involved in drug metabolism including cholangiocytes, liver or intestine, from patient-derived iPSCs (Takebe et al., 2013; Watson et al., 2014; Sampaziotis et al., 2015). Such a model will undoubtedly improve our understanding of the genetic basis and the molecular mechanisms that govern the side effects of drugs currently used for endometrial diseases.

## Hurdles and Challenges of Endometrial Organoids

The fact that endometrial organoids recapitulate the temporal progression of endometrial development presents both an advantage and disadvantage. While it offers an unprecedented opportunity for researchers to probe the different stages of human endometrial development, such organoids take a long time to proliferate and mature in culture, elevating cell culture costs and potentially compromising reproducibility (Table 1). The latter hurdle arises mainly from the self-organization property of organoids (more particularly for organoids derived from iPSCs). Therefore, researchers should carefully control the proliferation and maturation process of endometrial organoids, while also being critical in discriminating true phenotypes from observations of sporadic events in a dish. Poor control of this process will lead to an inherent variability between organoid samples, making it very difficult to quantitatively assess experiments in an unbiased manner. In order to understand the molecular and cellular mechanisms underlying endometrial development in physiological and pathological conditions, it is essential that every endometrial organoid display near-identical phenotypic characteristics in terms of size, shape, cellular composition, and architecture and similar functional properties.

Because of their 3D nature, the size of all organoids is limited by oxygen and nutrient supply. Viable parts of the organoids are restricted by the physical area over which oxygen and nutrients can diffuse from the surrounding media. Therefore, researchers should be aware of necrosis in the interior of organoids. Such a hurdle can be overcome introducing vascularization to the organoids. Thus, extensive effort will likely focus on the development of biomaterials and microfluidic systems that can be used to engineer a vascularized network to supply the organoid interior with sufficient oxygen and nutrients (Brassard and Lutolf, 2019).

While the endometrial organoid model is still in its infancy, a long term goal of this technology will be to apply it for cell replacement in regenerative medicine. However, significant improvements regarding the safety will be required in order to derive clinical grade iPSCs. An important concern would be selection of the optimal method for reprograming patient cells into iPSCs, as retroviral- or lentiviral-based methods can cause insertional inactivation of tumor suppressor genes, insertional activation of oncogenes and variability in the differentiation potential of these cells (for review see (Hibaoui and Feki, 2012, 2015). Among the integration-free methods of reprograming, episomal plasmids and Sendai viral vectors appear to be methods of choice for deriving clinical grade iPSCs.

## Concluding Remarks

Endometrial organoids, whether derived from primary human biopsy or from patient-derived iPSCs provide unprecedented opportunity to study the human endometrium. The possibility to derive endometrial and trophoblast organoids from primary biopsies (Boretto et al., 2017; Turco et al., 2017, 2018) together with the capacity to differentiate endometrial cells and trophoblast cells from iPSCs (Hibaoui and Feki, 2013; Horii et al., 2016; Miyazaki et al., 2018) offer new area of investigations. Such organoids can be exploited for disease modeling, drug screening, testing and benchmarking for novel therapeutics, as well as the potential evaluation of personalized therapeutic medicine approaches (Figure 1). Finally, considering the progression at which the biopsy-derived and iPSC-derived organoid field has advanced in the past 5 years (Nature Methods, 2018), there is a good reason for optimism that endometrial organoids will enhance our understanding of the molecular and cellular mechanisms involved in endometrial development and disease.

## Author Contributions

YH and AF conceived, designed, and wrote the manuscript.

### Conflict of Interest

The authors declare that the research was conducted in the absence of any commercial or financial relationships that could be construed as a potential conflict of interest.
